# HYPOPHOSPHATEMIC RICKETS: CASE REPORT

**DOI:** 10.1590/1984-0462/;2018;36;2;00009

**Published:** 2018-03-29

**Authors:** Marta Liliane de Almeida Maia, Ana Lucia Santos Abreu, Paulo Cesar Koch Nogueira, Maria Luiza Dautro Moreira do Val, João Tomas de Abreu Carvalhaes, Maria Cristina de Andrade

**Affiliations:** a Hospital Infantil Darcy Vargas, São Paulo, SP, Brasil.; b Universidade Federal de São Paulo, São Paulo, SP, Brasil.

**Keywords:** Rickets, hypophosphatemic, Hypophosphatemia, Fractures, Raquitismo hipofosfatêmico, Hipofosfatemia, Fraturas

## Abstract

**Objective:**

Early diagnosis and immediate treatment of hypophosphatemic rickets is of utmost importance as it may prevent subsequent sequelae. This report aims at warning pediatricians to consider the presence of the disease.

**Case description:**

Description of the metabolic profile, creatinine clearance, nutritional status, weight and body structure of a patient who presented the clinical-laboratorial characteristics of hypophosphatemic rickets and was followed in an outpatient clinic for tubulopathies over the period of 12 months. The patient had been bedridden for some time, was dependent on mechanical ventilation and presented an altered metabolic bone condition. Treatment was phosphate (initial: 65 mg/kg/day and final: 24,2 mg/kg/day), calcium (initial: 127 mg/kg/day, final: 48,4 mg/kg/day) and calcitriol (initial: 0.06 mcg/kg/day, final: 0.03 mcg/kg/day). The patient improved, evolving into spontaneous breathing and walking unaided. Laboratory results: calcium (mg/dL) initial 7.1, final 10.1; phosphate (mg/dL) initial 1.7 final 3.2; magnesium (mg/dL) initial 1.5 final 2.1, parathyroid hormone (pg/l) initial 85.8, final 52.7, alkaline phosphatase (UI/l) initial 12660, final 938; there was also improvement in weight/structural development (Z score: H/A initial: -6.05, final -3.64; W/A: initial -2.92, final -1.57) with presence of transitory gallstones. Creatinine clearance (mL/min/1.73m^2^bsa) was constant. The medication improved his laboratory results and nutritional status, but the patient did not return for two years for follow-up and, during this period, his condition has noticeably deteriorated.

**Comments:**

Early diagnosis and follow-up are essential in dealing with this pathology.

## RESUMO

**Objetivo:**

O raquitismo hipofosfatêmico precisa ser precocemente diagnosticado porque seu tratamento previne sequelas incapacitantes. Este relato alerta para a doença.

**Relato de caso:**

Relato de perfil metabólico, depuração de creatinina, estado nutricional e desenvolvimento pôndero-estatural de paciente com características clínico-laboratoriais de raquitismo hipofosfatêmico, atendido em ambulatório de tubulopatias por período de 12 meses. Chegou ao serviço após tempo prolongado acamado, dependente de ventilação mecânica e com perfil metabólico ósseo alterado. Terapêutica consistiu na administração de fósforo (inicial: 65 mg/kg/dia, final: 24,2 mg/kg/dia), cálcio (inicial: 127 mg/kg/dia, final: 48,4 mg/kg/dia) e calcitriol (inicial: 0,06 mcg/kg/dia, final: 0,03 mcg/kg/dia), e a análise constou da descrição das consultas, utilizando-se mediana de exames laboratoriais e dados antropométricos. Observou-se nítida melhora inicial do padrão respiratório do paciente, que evoluiu com ventilação espontânea e deambulação autônoma; com exames laboratoriais: cálcio (mg/dL) inicial 7,1, final 10,1; fósforo (mg/dL) inicial 1,7, final 3,2; magnésio (mg/dL) inicial 1,5, final 2,1; paratormônio (pg/L) inicial 85,8, final 52,7; fosfatase alcalina (UI/L) inicial 12660, final 938; e melhora do desenvolvimento pôndero-estatural (escore Z: E/I inicial: -6,05, final -3,64; P/I: inicial -2,92, final -1,57) com presença de litíase transitória. A depuração de creatinina (mL/min/1,73 m^2^sc) foi constante durante o seguimento. O tratamento propiciou benefícios clínicos, bioquímicos e nutricionais, mas, apesar da boa resposta inicial, a família abandonou o seguimento por dois anos, apresentando o paciente piora da deambulação e das deformidades esqueléticas.

**Comentários:**

Não apenas diagnóstico precoce é necessário, como também a adesão ao tratamento é fundamental para o sucesso na condução dessa patologia.

## INTRODUCTION

Rickets is characterized by the deficient mineralization of the growth plate, classified according to the scarcity of the prevalent mineral: calcium or phosphorus. In cases in which there is vitamin D resistance or deficiency, hypocalcemia associated with hypophosphatemia can be observed,^1^ and vitamin D deficiency is secondary or primary, as observed in nutritional rickets.

This condition, first considered as a change in the calcium and vitamin D metabolism only, gradually presented as a pathology characterized by the lack of availability of serum phosphate, necessary for the normal bone metabolism. There are some syndromes that present isolated renal phosphate loss, leading to hypophosphatemia, normocalcemia and primary rickets. According to the mode of inheritance, these syndromes are classified in: X-linked hypophosphatemic rickets, autosomal dominant and recessive hypophosphatemic rickets^1^ and hypophosphatemic rickets with hypercalciuria, being dependent or not of the fibroblast growth factor 23 (FGF23), a bone-derived hormone.^2^ ^,^ ^3^ FGF23 is a phosphate-regulating hormone that works on the kidney tubule cells, reducing the tubular reabsorption of phosphorus by blocking the sodium and phosphorus co-transporters (NaPi-IIa and NaPi-IIc) in the proximal tubule, which causes an increase in the urinary phosphate excretion. FGF23 also regulates 1-α-hidroxylase, inhibiting the activation of vitamin D 25OH to 1.25 (OH)_2_. The vitamin D, which is the most active form of vitamin D.^4^ The main differential diagnosis of hypophosphatemic rickets is nutritional rickets, in which it is possible to observe high rate of tubular phosphate reabsorption (RTPR) and the Fanconi syndrome, which is associated with metabolic acidosis and other serum and urinary electrolyte disorders.

Despite all of the genetic disorders described about rickets, the access to molecular investigation is not always fast and possible. However, the detailed physical examination, as well as the routine laboratory and radiological examinations, may guide the diagnosis, enabling the early treatment of the condition. The laboratory investigation consists on the evaluation of bone profile, with doses of total and ionic calcium, serum and urinary phosphate and RTPR, alkaline phosphatase, vitamin D and parathormone. The imaging examinations (X-ray) may show the presence of bone rarefaction, fractures, long bone arching, thoracic cage deformity and epiphyseal enlargement.

This study aimed at reporting a severe case of a patient with hypophosphatemic rickets, describing its nutritional and laboratory evolution during follow-up, showing the benefits of the therapy, which can be conducted even at the absence of a molecular diagnosis. This study was accepted by the Research Ethics Committee of Plataforma Brasil (n. 643.907), after the acceptance of the informed consent form.

## CASE REPORT

**Figure 1:**
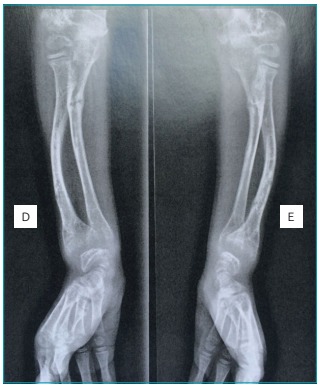
Forearm x-ray after the beginning of therapy.

Male patient, with history of bone deformities and recurrent respiratory infections that began at the age of nine months, with gradual regression of the neuropsychomotor development, losing acquisitions (crawling and sitting unassisted). The investigation of rickets began at the age of three, after a pathological humeral fracture. The initial examinations showed RTPR: 70.8% (normality value (NV): >80%), serum phosphate: 2.2 mg/dL (NV: 4-7), calcium: 7.5 mg/dL (NV: 8.6-10.2). The serum albumin level was normal, as well as the other electrolytes. It was not possible to dose vitamin D. Bone biopsy showed the presence of osteomalacia and the long bone X-ray showed hypomineralization and multiple consolidated fractures, diffuse osteopenia, epiphyseal dysplasia, podalic distal phalangeal hypoplasia (Figure 1). The kidney ultrasound showed changes in the echotexture of the renal parenchyma, ratifying the diagnosis of hypophosphatemic rickets, without family history.

Phosphate, calcium and calcitriol replacement began. Before the stabilization of the metabolic profile, at the age of five, the patient needed prolonged hospitalization in another service due to pneumonia, and was bedridden for 20 months. Tracheostomy with mechanical ventilation was required. He was assessed by a nephropediatrician, at the age of seven, and the drug therapy was readapted, which showed significant improvement in the clinical and laboratory scenarios. As observed in Table 1, in three months there was an improvement in the phosphate serum value (from 1.7 to 5.7 mg/DL), as well as calcium (from 7.1 to 9.6 mg/dL), alkaline phosphatase (12660 to 430 U/L) and parathormone (85.8 to 52.7 ng/L).

He received phosphorus replacement, 65 mg/kg/day, with tricalcium phosphate solution (5 mL=856 mg, in which 1 mL=34 mg of phosphorus and 63.2 mg of calcium), calcitriol, 0.06 mcg/kg/day (capsule with 0.25 mcg), and calcium 127 mg/kg/day, in the form of calcium carbonate (500 mg/capsule). The patient evolved with the normal respiratory pattern, enabling the tracheostomy decannulation process and ventilatory support. Regarding neuropsychomotor development, the patient, bedridden throughout the hospitalization period, started walking without support after three months of drug intervention and intensification of motor physical therapy. There was a progressive increase in the phosphorus drug replacement: 84.74 mg/kg/day, calcitriol: 0.063 mcg/kg/day and calcium: 169.5 mg/kg/day. During the 120-month follow-up, considering the period of therapy adjustment by the nephrologist as initial, the patient presented with gradual height improvement, passing from a height/age Z score of -6.05 to .3.64 (Table 2).

**Table 1:** Laboratory evolution of the studied patient, with initial values at the age of seven.

	Initial	3 m	6 m	12 m	Mean	Median	Minimum	Maximum	Reference value
Ca (mg/dL)	7.1	9.6	9.7	10.1	9.67	9.70	7.1	10.10	8.6-10.2
P (mg/dL)	1.7	5.7	4.6	3.2	3.70	3.20	1.70	5.70	4-7
Mg (mg/dL)	1.5	1.9	2.5	2.1	2.04	2.10	1.50	2.50	1.8-2.5
PTH (ng/L)	85.8	-	-	52.7	69.25	69.25	52.70	85.80	15-65
AF(U/l)	12660	430	642	938	3274	938	430	12660	<300
Cl.Cr	127	86.7	88.23	118.26	93.86	87.48	86.7	118.26	>90

m: months; Ca: calcium; P: phosphorus; Mg: magnesium; PTH: parathormone; AF: alcaline phosphatase; Cl.Cr: *estimated creatinine clearance.*

**Table 2:** Nutritional evolution of the studied patient, with initial values at the age of seven.

	Initial	3 m	6 m	12 m
Height/age Z-score	-6.05	-5.33	-4.75	-3.64
Weight/age Z-score	-2.92	-2.64	-2.55	-1.57
Weight/height Z-score	1.39	1.17	0.67	-
Body mass index	18.70	17.90	16.90	17.40
Body mass indez Z score	2.29	1.78	1.21	1.22

Z-score normality values: -2 to +2; m: months.

**Figure 2:**
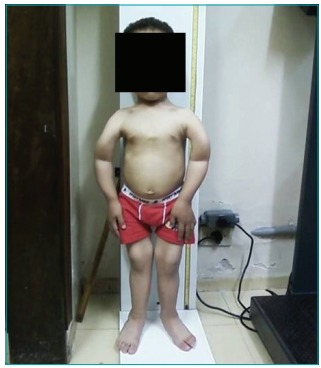
Clinical aspects of the reported patient after he began to walk.

In the last appointment of the regular visits, the patient did not present changes in gait; however, there were some skeletal changes: genu-varum, upper limb arching, Olympic forehead, bell-shaped thorax (Figure 2). At this time, the patient used calcitriol (0.03 mcg/kg/day), calcium (48.4 mg/k/day), and phosphorus replacement (24.2 mg/k/day). The initial creatinine clearance was 127 mL/min/1.73 m^2^ and the final was 118 mL/min/1.73 m^2^.

After the favorable response to the initial treatment, the patient missed the appointments for two years; when he came back, after this period, he could not walk, due to the worse condition of the skeletal deformities. At this time, he again presented with hypophosphatemia, increased levels of alkaline phosphatase and maintenance of phosphaturia.

## DISCUSSION

Even though the molecular diagnosis is important to classify rickets, the clinical manifestations and the laboratory changes were sufficient, in the patient reported, to diagnose the hypophosphatemic rickets, and guide the success of the therapy with consequent improvement of the general status and the bone and nutritional metabolic profile. It is worth to mention that during the abandonment of follow-up for two years, there was involution of motor skills and worsened bone deformities.

Approximately 90% of the filtered phosphate is reabsorbed in the proximal tubule, and the rest, in the distal tubule. In hypophosphatemia of extrarenal causes, less than 10% of the filtered phosphate is excreted in the urine. The luminal phosphate is reabsorbed in the proximal tubule by the electrogenic transporter NaPi-IIa (sodium-dependent phosphate transporter 2a), which transports three sodium ions for one phosphate, and one electroneutral transporter called NaPi-IIC (sodium-dependent phosphate transporter 2c), which transports two sodium molecules for each phosphate.^5^

The bone irregularities of the patient were a result of prolonged hypophosphatemia, since it is known that the maintenance of the levels of intra and extracellular phosphate inside a narrow band is important for several biological processes, including the energy metabolism, skeletal development and bone integrity. Besides, phosphorus deficiency can compromise chondrocyte maintenance, causing the block of bone neoformation, resulting in delayed growth and rickets,^6^ therefore justifying the weight-height delay in the reported patient.

The presence of phosphate in normal levels is also essential for the occurrence of bone mineralization, and when it lacks, it can lead to the onset of osteomalacia,^6^ observed in this patient’s biopsy. In hypophosphatemic rickets, serum calcium is usually normal or slightly reduced. In this specific case, other factors may have influenced bone metabolism, for instance, vitamin D deficiency because the patient was bedridden, with low exposure to the sun, as well as the prolonged immobility, which is bad for bone mineralization.

Hypophosphatemic rickets is among the differential diagnoses of rickets in childhood, and its initial therapy is composed of phosphorus and calcitriol replacement. The improvement in growth can be observed in the first year of therapy, especially in prepubescent children. In the case described, there was gradual improvement in height, without considerable increase in the body mass index (BMI), due to the important height recovery.

The patient presented with transient renal lithiasis during follow-up (calculus measuring 0.35 cm in the kidney upper calyx), complication reported by other authors resulting from the instituted therapy. The supplementation with calcium, phosphate and calcitriol can lead to the onset of renal lithiasis. This complication occurs due to hypercalcemia, hypercalciuria, and hyperparathyroidism secondary to transient hyperphosphatemia,^7^ highlighting the need to monitor calciuria when using active vitamin D. Besides, hypophosphatemia can be related with lithiasis and nephrocalcinosis, in case the patient presents with hyperphosphaturia or hypercalciuria.

The patient did not receive the maximum dose of phosphate (90.0 mg/kg/day) due to the onset of renal lithiasis. This supplementation, which was not yet ideal (24.2 mg/kg/day), could be one of the causes of hypophosphatemia at the end of one year of follow-up, besides phosphaturia itself.

The most important differential diagnoses to be considered are the Fanconi syndrome and nutritional rickets. The Fanconi syndrome shows metabolic acidosis, hypouricemia, proteinuria and serum and urinary changes of other electrolytes, besides episodes of recurrent dehydrations. Nutritional rickets shows changes in bone metabolism, however, the rate of the tubular reabsorption of phosphate is close to 100%. These diseases were ruled out, since the patient did not present with acid-base disorders nor proteinuria; urinary phosphate loss associated with hypophosphatemia was observed.

Even though the diagnosis and initial treatment of hypophosphatemic rickets can be conducted without molecular diagnosis, the detailed diagnosis obtained by the genetic investigation is important, mainly for genetic counseling.

X-linked hypophosphatemic rickets, described in 1958, is the most common form of primary rickets, with incidence of 1:20,000.^8^ It is characterized by a flaw in the proximal tubular reabsorption of phosphate, secondary to the mutation in the phosphate-regulating gene with homologies for endopeptidases in chromosome X (PHEX). This gene codifies an endopeptidase that degrades and inactivates hormone-like substances called phosphatonin (proteins in the FGF family).^9^ With the mutation in gene PHEX, the degradation and inactivation of FGF reduces, resulting in the increased excretion of phosphate and in the compromise of bone mineralization.^3^ ^,^ ^10^ ^,^ ^11^

Autosomal dominant hypophosphatemic rickets, described in 1971,^8^ occurs because of mutations in the gene of FGF23 in chromosome 12p13, with gain in function, resulting in high levels of FGF23. FGF23,besides inhibiting the reabsorption of renal phosphate, also stops the synthesis of calcitriol, active form of vitamin D.^12^ ^,^ ^13^ ^,^ ^14^ In this type of rickets, there is phosphaturia, 1.25 (OH)_2_, normal or reduced serum vitamin D3, and skeletal changes that are typical of this pathology, such as fractures, rachitic rosary and/or osteomalacia.^8^

The autosomal recessive hypophosphatemic rickets (ARHR), without hypercalciuria, is characterized by showing isolated renal phosphate loss. It is subdivided in three subtypes: ARHR1, caused by mutations that inactivate gene DMP1, which codifies the dentin matrix protein; ARHR2, caused by a mutation that inactivates gene ENPP1, which codifies the ectonucleotide pyrophosphate/ phosphodiesterase 1; and RHAE3, recently described in a family from Norway, which presents association between biallelic mutations in a family with 20 sequence similarities (FAM20C, which codifies a major protein in processes of phosphorylation), and FGF23,^4^ causing the inactivation of FAM20C and increased levels of FGF23. The three forms of mutation lead to an increase in the expression of FGF23 and flaws in the maturation of the osteocyte.^4^ ^,^ ^15^

The hereditary hypophosphatemic rickets with hypercalciuria is characterized by mutations in the gene of the sodium and phosphorus co-transporters (SLC34A3), which leads to severe dysfunctions in the NaPi-IIa co-transporter.^16^ The clinical Picture is manifested by rickets and/or osteomalacia. Lighter forms can be underdiagnosed. This form is different from those described previously for presenting normal or high concentrations of calcitriol for the reduced level of hypophosphatemia and FGF23. Hypercalciuria is probably owed to the high level of calcitriol, with consequent increase in the intestinal calcium absorption.^17^ ^,^ ^18^

Besides these syndromes, there are other scenarios that can be manifested with hypophosphatemic rickets, described as follows:

- Mutations in the sodium-hydrogen exchanger regulatory factor (NHERF1), which regulates the activity of the sodium-phosphate co-transporter and is related with phosphaturia and hypophosphatemia;^16^
- Tumor-induced osteomalacia (paraneoplastic disease), which are mesenchymal tumors and produce excessive phosphaturic peptides.

The conclusion is that the earlier the diagnosis, the lower the sequelae resulting from the lack of these minerals, despite the probability of forming calculi. The regular follow-up with adequate use of medications is mandatory to maintain the acquired acquisitions. Regarding chronic and incurable diseases, with clinical manifestations that started in childhood, diagnosis and intervention are important for reducing the morbimortality of these patients.
